# Modeling arbitrarily applicable relational responding with the non-axiomatic reasoning system: a Machine Psychology approach

**DOI:** 10.3389/frobt.2025.1586033

**Published:** 2025-09-22

**Authors:** Robert Johansson

**Affiliations:** Department of Psychology, Stockholm University, Stockholm, Sweden

**Keywords:** artificial general intelligence (AGI), arbitrarily applicable relational responding, operant conditioning, Non-Axiomatic Reasoning System (NARS), machine psychology, adaptive learning

## Abstract

Arbitrarily Applicable Relational Responding (AARR) is a cornerstone of human language and reasoning, referring to the learned ability to relate symbols in flexible, context-dependent ways. In this paper, we present a novel theoretical approach for modeling AARR within an artificial intelligence framework using the Non-Axiomatic Reasoning System (NARS). NARS is an adaptive reasoning system designed for learning under uncertainty. We introduce a theoretical mechanism called *acquired relations*, enabling NARS to derive symbolic relational knowledge directly from sensorimotor experiences. By integrating principles from Relational Frame Theory—the behavioral psychology account of AARR—with the reasoning mechanisms of NARS, we conceptually demonstrate how key properties of AARR (mutual entailment, combinatorial entailment, and transformation of stimulus functions) can emerge from NARS’s inference rules and memory structures. Two theoretical demonstrations illustrate this approach: one modeling stimulus equivalence and transfer of function, and another modeling complex relational networks involving opposition frames. In both cases, the system logically demonstrates the derivation of untrained relations and context-sensitive transformations of stimulus functions, mirroring established human cognitive phenomena. These results suggest that AARR—long considered uniquely human—can be conceptually captured by suitably designed AI systems, emphasizing the value of integrating behavioral science insights into artificial general intelligence (AGI) research. Empirical validation of this theoretical approach remains an essential future direction.

## 1 Introduction

Human intelligence is marked by an extraordinary capacity for symbolic reasoning—the ability to understand and manipulate symbols (words, ideas, abstract concepts) and their relationships in a flexible manner. An aspect of this flexibility is the capability to derive new relationships between symbols without direct training, purely based on their contextual relations. In cognitive and behavioral psychology, this phenomenon is captured by the concept of Arbitrarily Applicable Relational Responding (AARR), which underlies human language and higher cognition (Hayes et al., 2001; Hayes et al., 2021). AARR refers to the learned behavior of relating stimuli in arbitrary ways (not dictated by the physical properties of the stimuli, but by contextual cues and social learning). For example, once a child learns that the spoken word “dog” refers to an actual furry pet, the child responds to the word as if it is functionally equivalent to the animal itself—experiencing excitement or happiness when hearing the word, similar to encountering the dog. Such symbolic equivalence is not determined by physical similarity but by relational learning. Derived relational responding of this type is considered a hallmark of human language and reasoning, enabling everything from understanding metaphors to performing complex analogies.

While humans readily perform AARR, instantiating this ability in artificial intelligence (AI) systems remains a formidable challenge. Traditional symbolic AI systems typically rely on explicitly programmed logic rules or axioms, and machine learning systems (like deep neural networks) often require vast amounts of data and struggle with extrapolating knowledge in the absence of direct examples. Achieving human-like symbolic reasoning in a machine calls for an approach that can learn relational patterns from a few examples and generalize them in a context-sensitive way, much as humans do. In other words, we seek an AI that can learn how to relate rather than being pre-programmed with all possible relations.

In this paper, we propose that AARR can be effectively modeled within a particular AI framework known as the Non-Axiomatic Reasoning System (NARS). NARS is an AI reasoning architecture designed to operate under the real-world constraints of insufficient knowledge and resources (i.e., it does not assume a closed, complete set of axioms or unlimited processing power) (Wang, 2013; Wang, 2022). Instead of a fixed logic, NARS uses an adaptive logic (Non-Axiomatic Logic, NAL) that allows it to learn from experience, update its beliefs probabilistically, and make plausible inferences even when knowledge is incomplete. These features make NARS a strong candidate for modeling the emergent, learned relations that characterize AARR.

The key contribution of this work is to demonstrate a computational method for describing human-like symbolic reasoning (AARR) in a machine by utilizing NARS’s capabilities. We integrate theoretical insights from Relational Frame Theory (RFT) (Hayes et al., 2001; Hayes et al., 2021) — the behavioral theory that explicates AARR—with the algorithmic machinery of NARS. We propose a novel theoretical mechanism called *acquired relations*, enabling NARS to derive symbolic relational knowledge directly from sensorimotor experiences. In doing so, we show that an AI system can learn and derive relationships among symbols in a manner analogous to human relational learning. This integration provides a framework for studying and implementing cognitive phenomena like language and abstract reasoning in AI. Importantly, our approach goes beyond purely mechanistic or narrow AI methods: rather than training a black-box neural network on vast relational datasets, we employ a functional approach grounded in how relations are learned and used by humans (Johansson, 2024a). This allows the system to capture the contextual control and generalizability of human relational responding.

This integrative approach aligns with the broader interdisciplinary perspective of *Machine Psychology* (Johansson, 2024a; Johansson, 2024b), which systematically applies principles from learning psychology—such as operant conditioning, generalized identity matching, and functional equivalence—to artificial intelligence architectures, aiming to replicate increasingly complex cognitive phenomena in machines (See Table 1 for an overview of how the present research fits with previously conducted studies).

**TABLE 1:** Overview of psychological processes, NARS mechanisms, layers (from Wang, 2013), and references.

Psychological process	NARS mechanisms	NARS layers	References
Operant conditioning	Temporal reasoning and procedural reasoning	7–8	(Johansson, 2024b)
Generalized identity matching	+Abstraction	+6	(Johansson et al., 2023)
Functional equivalence	+Implications	+5	(Johansson et al., 2024)
Arbitrarily applicable relational eesponding	+Acquired relations	+4	This study

We validate our approach with two experimental paradigms inspired by human studies. The first is a stimulus equivalence task involving three groups of stimuli and tests for derived symmetric and transitive relations, as well as a demonstration of the transformation of stimulus function (e.g., if one stimulus in a set is given a certain meaning or consequence, the others derived to be equivalent to it also reflect that meaning) (Hayes et al., 1987). The second is an oppositional relational network task, where the system learns a network of “opposite” relations (a case of a more complex relational frame) and we examine how this leads to emergent relations and transformations of function consistent with what is observed in human experiments on relational framing of opposites (Roche et al., 2000).

The remainder of this article is organized as follows. Section 2 provides background on Arbitrarily Applicable Relational Responding, the Non-Axiomatic Reasoning System, and our research approach—Machine Psychology. Section 3 reviews related work, contrasting our perspective with other AI and cognitive modeling efforts. Section 4 introduces our theoretical framework, explaining how acquired relations enable modeling of AARR within NARS. Section 5 outlines the methodology behind our illustrative theoretical demonstrations, and Section 6 summarizes their key results, with detailed conceptual derivations provided in the Supplementary Material. Finally, Section 7 discusses broader implications for artificial general intelligence and cognitive science, and outlines directions for future empirical research. Collectively, these contributions establish a theoretical foundation for the empirical study of relational responding in adaptive AI systems.

## 2 Theoretical background

### 2.1 Arbitrarily applicable relational responding

Arbitrarily Applicable Relational Responding (AARR) is a concept from behavioral psychology that refers to a general pattern of learned behavior: responding to the relation between stimuli rather than just the stimuli themselves, and doing so in a way that is not determined by the stimuli’s physical properties but by contextual cues and history of reinforcement (Hayes et al., 2001; Hayes et al., 2021). This idea is central to Relational Frame Theory (RFT), a modern behavioral theory of language and cognition (Hayes et al., 2001; Hayes et al., 2021). According to RFT, virtually all of human language and higher cognition is founded upon AARR—the ability to treat different stimuli as related along various dimensions (e.g., *same*, *different*, *greater than*, *opposite*, etc.) purely as a result of learned context, not because of any inherent relationship in their physical features.

Three key properties define AARR and distinguish it from simple associative learning.

1. Mutual Entailment: This is the bidirectionality of derived relations. If a person learns a relation in one direction (e.g., A is larger than B), they can derive the relation in the opposite direction (B is smaller than A) without direct training (Luciano et al., 2007). In classical terms, mutual entailment encompasses symmetric relations (if A=B , then B=A ) and the inverses of asymmetrical relations (if A>B , then B<A ) in a generalized way. Notably, the derived relation might not be identical in form (for instance, *larger than* vs *smaller than* are inverse relations rather than exactly the same), but they are mutually implied by each other given the contextual cues (such as the contextual cue for comparison).
2. Combinatorial Entailment: This is the ability to derive new relations from combinations of learned relations. For example, if one learns that A is related to B, and B is related to C, one can often derive a relation between A and C, depending on the nature of the relation. In the simplest case, if A=B and B=C (coordination relations), then one can derive A=C (equivalence). If A>B and B>C (a comparative relation of “more than”), one can derive A>C (“A is more than C”). These are akin to transitive inferences, but RFT uses the term *combinatorial* entailment to emphasize that the new relation emerges from the combination of two or more other relations.
3. Transformation of Stimulus Function: Perhaps the most distinctive aspect, this refers to the way the functions of stimuli (their meaning, emotional valence, or behavioral effects) can change based on the relations they participate in (Dymond and Rehfeldt, 2000). In other words, if two stimuli are related in a certain way, any psychological function attached to one stimulus (like being pleasant, having a certain name, evoking a specific response) can be transferred to the other stimulus in accordance with their relation. For instance, suppose a person is taught that stimulus A is equivalent to stimulus B ( A=B , a coordination relation), and separately, stimulus A acquires a particular function (e.g., A is paired with a reward or labeled as “good”). Then, without additional training, the person may treat stimulus B as also having that function (finding B pleasant or “good”), because B is in the same equivalence class as A. If the relation is one of opposition, the functions might transfer in an opposite manner (e.g., if A is opposite to B, and A is associated with “good,” B might be seen as “bad”) (Roche et al., 2000). Transformation of function demonstrates how relational learning can govern the meaning of symbols in context.

An example can illustrate these principles. Imagine a scenario in a coffee shop: A newcomer is told that “Espresso is stronger than Americano, and Americano is stronger than Caffé au Lait.” From just this information, the person can derive that Espresso is stronger than Caffé au Lait, and conversely, Caffé au Lait is weaker than Espresso (combinatorial entailment and mutual entailment for the comparative frame). Now, suppose the person actually tastes an Americano and finds it strong and bitter. That experience may attach a function (strong flavor) to Americano. Due to the relational network, the person might now expect that Espresso (which was said to be stronger than Americano) has an even stronger taste, and that Caffé au Lait (weaker than Americano) has a milder taste, even though they have never tasted Espresso or Caffé au Lait. This is a transformation of stimulus function across a comparative relation network: the direct experience with one item (Americano) transformed the anticipated qualities of the related items (Espresso, Caffé au Lait) in line with the learned relations.

Relational Frame Theory has identified numerous types of relational patterns (called *relational frames*) that humans can learn. Some prominent examples include frames of *coordination* (equivalence/sameness), *distinction* (different from), *comparison* (more than/less than as in the coffee strength example), *opposition*, *hierarchy* (e.g., category membership relations, like “X is a kind of Y”), *temporal* (before/after), *spatial* (here/there), and *deictic* (I/you, now/then, here/there, which involve perspective) (Hayes et al., 2001; 2021). All these frames share the properties of mutual and combinatorial entailment and can lead to transformations of function, though the exact nature of the entailments depends on the frame.

It is important to note that AARR is considered an *operant behavior*, meaning it is learned through a history of reinforcement and context, rather than being an innate or automatic reflex (Hayes et al., 2021). Crucially, according to RFT, derived relational responding (such as mutual entailment, combinatorial entailment, and transformation of function) is established via *multiple exemplar training (MET)*, a well-documented learning process through which individuals are exposed to a variety of relational examples until relational responding generalizes to new, untrained examples without direct reinforcement (Luciano et al., 2007; Hayes et al., 2021). Thus, explicitly training relational patterns initially is fully consistent with RFT, and subsequent relational responding is considered “emergent” precisely because it generalizes beyond reinforced examples due to this learning history. The term “arbitrarily applicable” emphasizes that any stimuli, regardless of their formal properties, can be related in any way, given the appropriate training context. Humans, especially those with language ability, seem uniquely capable of this kind of learning (Devany et al., 1986). Indeed, research has shown that stimulus equivalence (a basic form of AARR focusing on sameness) reliably appears in humans but not in most non-human animals without language training, with only rare exceptions (Schusterman and Kastak, 1993). This link between language and AARR suggests that a capacity for relational responding is a defining feature of higher cognition.

Relational Frame Theory provides a perspective on general intelligence as well. Rather than viewing intelligence as a monolithic IQ or a fixed set of problem-solving abilities, RFT suggests intelligence involves a rich repertoire of relational skills (Cassidy et al., 2016; Hayes et al., 2021). From this viewpoint, improving one’s ability to learn and manipulate complex relational networks should enhance cognitive performance. Studies have found that training individuals on relational tasks can increase scores on standard intelligence tests (Cassidy et al., 2016). Programs like *SMART* (Strengthening Mental Abilities with Relational Training) and *PEAK* (Promoting the Emergence of Advanced Knowledge) aim to boost cognitive and language abilities by systematically exercising relational responding abilities (Dixon et al., 2017).

In summary, AARR, as characterized by RFT, captures the flexibility, generativity, and context-sensitivity of human symbolic reasoning. Modeling this phenomenon in an AI system requires that the system can represent relations between symbols, infer new relations from old, and dynamically update what symbols mean based on relational context. Next, we discuss NARS, which we propose as a suitable candidate for this challenge.

### 2.2 Non-Axiomatic Reasoning System (NARS)

The Non-Axiomatic Reasoning System (NARS) is an AI system and cognitive architecture developed by Pei Wang (Wang, 2013; Wang, 2022) with the goal of realizing a form of general intelligence that operates effectively under real-world constraints. The name “non-axiomatic” reflects that NARS does not assume a predefined, complete set of axioms or truths about the world; instead, it must learn and reason non-axiomatically, meaning all its knowledge is gleaned from experience and is always revisable. NARS was built on the recognition that an intelligent agent in the real world must cope with insufficient knowledge and insufficient resources (a principle Wang abbreviates as AIKR: Assumption of Insufficient Knowledge and Resources (Wang, 2019)). Unlike classical logic systems that are brittle outside of their given axioms, NARS is adaptive and is constantly updating its beliefs and strategies as new information comes in, somewhat akin to a human continually learning and adjusting their understanding.

At the core of NARS is an AI reasoning framework called Non-Axiomatic Logic (NAL). NAL is a formal logic that extends term logic (a kind of logic dealing with relationships between terms or concepts) and is probabilistic in nature. NARS uses an internal language, Narsese, to represent knowledge. All pieces of knowledge in NARS are expressed as statements in Narsese, which typically have a subject and a predicate and a copula connecting them (the copula defines the type of relation between subject and predicate). The simplest form is an inheritance relation “ S→P ” meaning “ S is a kind of P ” or “ S implies P ” in a category sense. For example, one could represent “Tweety is a bird” as Tweety→Bird , and “Birds are animals” as Bird→Animal . NAL can then derive Tweety→Animal by inference (a kind of syllogism) (Wang, 2013). In addition to inheritance, Narsese includes other basic copulas such as similarity (noted as ↔ in Narsese, meaning two terms are similar or equivalent in some sense), implication ( → with different context indicating temporal or causal implication), and equivalence ( ⇔ for bi-conditional statements). Through combinations of these, NARS can represent a wide variety of knowledge, including rules like “if X happens then Y tends to happen” (an implication), or “Concept A is similar to Concept B” (a similarity statement).

Crucially, every statement in NARS carries a measure of uncertainty. NARS does not use binary true/false assignments; instead, each piece of knowledge has a truth value with two parameters: *frequency* (a measure akin to probability based on how often the relation has been true in experience) and *confidence* (reflecting the amount of evidence available) (Hammer, 2022; Wang, 2022). This allows NARS to reason under uncertainty and update its beliefs as new evidence arrives. For example, if initially NARS has little evidence about “Tweety can fly,” it might assign it a low confidence. If many observations confirm it, the confidence (and perhaps the frequency) increases. See the Supplementary Material for more information regarding frequency and confidence.

Another distinguishing feature of NARS is its approach to resource constraints. NARS operates in real-time and has a limited “budget” for attention and memory. It cannot consider all knowledge all the time. Instead, it uses a priority mechanism to decide which tasks (questions, goals, new knowledge) to process next, based on factors like urgency and relevance. This ensures that at any given moment, the system focuses on the most pertinent information, allowing it to scale to larger problems by not getting bogged down in less relevant details.

Recent implementations of NARS include OpenNARS and specifically a variant called OpenNARS for Applications (ONA) (Hammer and Lofthouse, 2020). ONA is tailored for integration into practical applications, including robotics. It extends the basic NARS framework with sensorimotor capabilities, meaning it can handle input from sensors and send output to actuators (motors) as part of its reasoning. This is done by treating sensorimotor events also as terms in the language (for instance, a sensory observation or a motor command can be a term that participates in statements). In ONA, the reasoning engine is capable of doing temporal inference, understanding sequences of events and causality. Temporal relations in Narsese might be represented with additional notation - for example, A⇒B might denote events A and B happening in sequence. ONA’s design includes components like event buffers, concept memory, and distinct inference processes for different types of tasks (e.g., some for immediate reactions, some for long-term learning) (Hammer and Lofthouse, 2020; Hammer, 2022).

For the purposes of this work, what is important is that NARS (and ONA) provides.

1. A flexible knowledge representation that can express arbitrary relations between symbols (via terms and copulas in Narsese).
2. Inference rules that can derive new relationships from known ones, analogous to the entailments described in RFT. For example, NARS can perform syllogistic inference (if A→B and B→C , derive A→C ) and inductive inference (generalizing or specializing relations based on evidence), which parallel combinatorial entailment in AARR.
3. The ability to incorporate new knowledge on the fly and revise existing knowledge, which is essential for any learning system attempting to acquire relational behavior through training.
4. The ability to handle context and switch between tasks, somewhat akin to how contextual cues in AARR determine which relation applies. In NARS, context is handled through its concept activations and the specific questions posed to the system; it is not identical to the notion of contextual cues in RFT, but NARS can take context into account by treating it as just another piece of information in the premise of a statement or rule.

In short, NARS can be seen as a unified cognitive model that does not separate reasoning, learning, memory, and perception into different modules; the same underlying logic and control mechanism handles all these functions (Wang et al., 2022). This makes it very appealing for modeling complex cognitive phenomena like AARR, because we do not need to bolt together separate systems for learning relations and for reasoning about them—NARS does both in one framework. The challenge is to design the right way to present relational training to NARS and possibly to extend NARS with any additional mechanisms so that it can exhibit mutual and combinatorial entailment and transformation of functions in a manner comparable to humans.

### 2.3 Machine psychology: bridging learning psychology and adaptive AI

Machine Psychology is an interdisciplinary framework that integrates learning psychology with adaptive AI systems, such as NARS, to explore the emergence of cognitive behaviors in artificial agents (Johansson, 2024a; Johansson, 2024b). This approach systematically investigates increasingly complex learning processes, drawing from operant conditioning, generalized identity matching, and functional equivalence, which are fundamental to relational cognition. In Table 1, we clarify how this systematic approach has been carried out in previous studies.

In this work, we assume that the system is interacting with the environment using different sensors. A key sensor that will be used throughout the entire paper is the assumption of a location sensor. Objects perceived by the vision system would using this model all be assigned a location. The labels sample, left, right, etc., are totally arbitrary. They are chosen by the designer and are only labels used to indicate that different objects are perceived at different locations.

**FIGURE 1:**
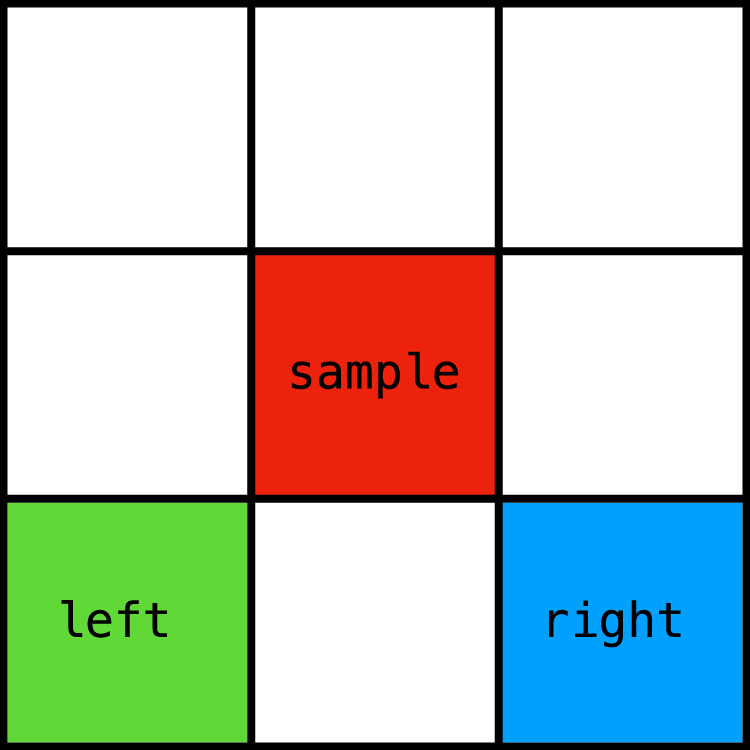
An example scene where the system perceives three different colors at three different locations.

We could also imagine that the system is equipped with a color sensor, and is interacting with a Matching-to-sample procedure. For example, as illustrated in Figure 1, something red is in the sample position, something green is to the left, and something blue to the right. This could be described that the only “eyes” that the system have are location and color, meaning that other object properties like shape and size couldn’t be perceived by that system.

The way we represent such interactions with the world in this paper is like the following:

<(sample ∗ red) --> (loc ∗ color)>. :|:

<(left ∗ green) --> (loc ∗ color)>. :|:

<(right ∗ blue) --> (loc ∗ color)>. :|:

The scene is described by two temporal statements (as indicated by:—:). Perceiving a green object to the left can be described as an interaction between perceiving to the left, and perceiving green. Hence, the statement <(left ∗ green) --> (loc ∗ color)> can be seen as a composition of <left --> loc> and <green --> color>. This encoding of object properties at certain locations will be used throughout this paper. Importantly, also an OCR detector will be assumed in the experiments carried out in the present study, leading to interactions as the one illustrated below.

<(sample ∗ A1) --> (loc ∗ ocr)>. :|:

<(left ∗ B1) --> (loc ∗ ocr)>. :|:

<(right ∗ B2) --> (loc ∗ ocr)>. :|:

For details regarding the experimental setup used in the research described below, see the section in the Supplementary Material, that clarifies Narsese syntax and key concepts.

#### 2.3.1 Operant conditioning with NARS

The foundation of Machine Psychology is built on operant conditioning, a fundamental demonstration of adaptive behavior (Johansson, 2024b). In our research, NARS was exposed to operant contingencies where behaviors were reinforced based on temporal and procedural reasoning. This enabled NARS to learn through interaction with its environment, adjusting actions based on feedback, similar to how organisms learn in response to consequences. The results demonstrated that NARS could acquire and refine behaviors through reinforcement, providing an essential basis for more advanced relational learning.

<(left ∗ blue) --> (loc ∗ color)>. :|:

<(right ∗ green) --> (loc ∗ color)>. :|:

G! :|: *// Establish G as a goal*

*// Executed with motor babbling:*

*// ^select executed with args (* *{SELF}∗ right)*

*G. :|: // Provide G as a consequence*

*// Derived with frequency 1, and confidence 0.19:*

*// <(<(right ∗ green) --> (loc ∗ color)> &/ <(* *{SELF} ∗ right) --> ^select>) =/> G>.*

#### 2.3.2 Generalized identity matching with NARS

Building upon operant conditioning, our research extended into generalized identity matching, which involves recognizing and responding to identity relations across varying stimuli (Johansson et al., 2023). This required NARS to utilize complex learning mechanisms, including abstraction and relational generalization. By introducing an abstraction mechanism to NARS, we enabled it to derive identity relations beyond explicit training examples, mirroring human cognitive abilities in symbolic matching tasks. The results showed that NARS could generalize identity relations to novel stimuli, demonstrating an emergent form of relational reasoning.

Let’s say that the system was exposed to the following NARS statements in the training phase.

<(sample ∗ blue) --> (loc ∗ color)>. :|:

<(left ∗ green) --> (loc ∗ color)>. :|:

<(right ∗ blue) --> (loc ∗ color)>. :|:

G! :|:

NARS could execute match with sample and right (from motor babbling or a decision based on previous experience), which would be considered correct, and hence the feedback G. :— : would be given to NARS, followed by 100 time steps. Only from this single interaction, NARS would form both a specific and a general hypothesis. 

<((<(sample ∗ blue) --> (loc ∗ color)> &/

<(right ∗ blue) --> (loc ∗ color)>) &/

<( {SELF} ∗ (sample ∗ right)) --> ^match>) =/> G>

*// frequency: 1.00, confidence: 0.15*

<((<(#1 ∗ #2) --> (loc ∗ color)> &/

<(#3 ∗ #2) --> (loc ∗ color)>) &/

<( {SELF} ∗ (#1 ∗ #3)) --> ^match>) =/> G>

*// frequency: 1.00, confidence: 0.15*

#### 2.3.3 Functional equivalence with NARS

Further advancing Machine Psychology, we explored functional equivalence, a process in which stimuli become interchangeable in guiding behavior due to shared functional properties (Johansson et al., 2024). This study introduced additional inference mechanisms into NARS, allowing it to derive new relations based on implications and acquired equivalences. Functional equivalence is critical for understanding how abstract categories are formed and used in problem-solving. Our findings indicate that NARS can establish and apply functional equivalence relations, effectively transferring learned functions between distinct but related stimuli.

<(s1 ∗ A1) --> (loc ∗ ocr)>. :|:

G! :|:

*// Executed with motor babbling*

<( {SELF} ∗ R1) --> ^press>. :|:

G. :|:

*// Derived*

<(<(s1 ∗ A1) --> (loc ∗ ocr)> &/

<( {SELF} ∗ R1) --> ^press>) =/> G>.

100

<(s1 ∗ A2) --> (loc ∗ ocr)>. :|:

G! :|:

*// Executed same operation with motor babbling*

<( {SELF} ∗ R1) --> ^press>. :|:

G. :|:

*// Derived*

<(<(s1 ∗ A2) --> (loc ∗ ocr)> &/

<( {SELF} ∗ R1) --> ^press>) =/> G>.

Since the system derived two contingencies that only differed in the pre-condition, statements like the following (functional equivalence) would also be derived.

<<($1 ∗ A1) --> (loc ∗ ocr)> ==> <($1 ∗ B1) --> (loc ∗ ocr)>>.

<<($1 ∗ B1) --> (loc ∗ ocr)> ==> <($1 ∗ A1) --> (loc ∗ ocr)>>.

These studies collectively illustrate the progression from simple operant conditioning to complex relational cognition, reinforcing Machine Psychology as a viable framework for advancing artificial general intelligence (AGI). An overview of the systematic approach Machine Psychology has taken, can be seen in Table 1. By systematically integrating behavioral learning principles with adaptive AI reasoning, this approach contributes to the development of more flexible, human-like intelligence in machines.

## 3 Related work

Integrating principles of human cognition and learning into AI systems is a growing interdisciplinary endeavor. However, Relational Frame Theory (RFT) and its core concept of Arbitrarily Applicable Relational Responding (AARR) have seen relatively little application in mainstream AI research. Most approaches to relational reasoning in AI have taken alternative paths.

### 3.1 Symbolic AI and knowledge graphs

Traditional symbolic reasoning systems, such as knowledge graph inference engines and logic-based AI, typically represent relations axiomatically (Lenat, 1995; Rosenbloom et al., 2016). These systems utilize explicitly predefined relational structures (e.g., ontological relationships like “isFatherOf” being inverse to “isChildOf”). They do not usually learn these relations dynamically but rely instead on manually crafted knowledge. In contrast, the proposed NARS-based approach aims at learning arbitrary relations from experience, enabling dynamic derivation of novel relations without predefined axioms.

### 3.2 Machine learning for relational tasks

In the machine learning domain, methods such as relational reinforcement learning, graph neural networks, and transformer-based models excel at extracting patterns from relational datasets. For example, DeepMind’s Relation Networks can effectively learn relational structures to answer visual-spatial questions from large-scale training data (Santoro et al., 2017). However, these data-driven methods typically require substantial training examples and may not guarantee key relational properties such as mutual or combinatorial entailment. Furthermore, these methods often lack interpretability and struggle with few-shot generalization—a core strength of human cognition that NARS aims to model by deriving relational structures adaptively from minimal and context-sensitive experiences.

### 3.3 Bayesian approaches to relational learning

Bayesian methods, including probabilistic programming and Bayesian relational modeling, represent relational structures while also modeling uncertainty (Nitti et al., 2016; Tenenbaum et al., 2011). These approaches are highly effective in generalizing from limited data, but they typically depend on predefined model structures and well-defined priors. As a result, dynamically deriving novel relational structures purely from interaction or flexibly adapting to context-sensitive relations can be challenging. By contrast, our NARS-based framework inherently constructs relational structures directly from interaction and accommodates dynamic, context-dependent inference without reliance on extensive predefined priors.

### 3.4 Statistical relational learning and neurosymbolic AI

Recent advances in Statistical Relational Learning (SRL) and neurosymbolic AI methods integrate symbolic logic with statistical and neural learning techniques (Marra et al., 2024). These hybrid methods effectively handle relational inference tasks by leveraging symbolic representation and data-driven learning. However, SRL methods typically require large datasets and predefined structures, potentially limiting their adaptability in low-data or dynamically evolving contexts. Our approach utilizing NARS offers a complementary perspective by emphasizing adaptive reasoning and minimal-data learning, targeting scenarios that demand rapid relational inference from limited interactions.

### 3.5 Inductive logic programming

Inductive Logic Programming (ILP) is another well-established paradigm for symbolic relational learning, focusing on deriving relational rules from structured data (Cropper and Dumančić, 2022). Recent ILP applications have successfully modeled cognitive processes in robotic systems, enabling robots to generalize relational tasks from expert feedback (Meli and Fiorini, 2025). While powerful, ILP generally relies on explicitly defined logical frameworks and structured training examples. In contrast, our proposed integration of NARS and RFT uniquely emphasizes adaptive, context-sensitive relational learning, minimizing reliance on predefined logic templates or extensive datasets.

### 3.6 Computational approaches inspired by RFT

Few computational approaches explicitly model AARR as defined by RFT. Early computational models attempted to simulate stimulus equivalence and relational responding through neural network approaches (Barnes and Hampson, 1993; Cullinan et al., 1994). These connectionist methods successfully modeled basic relational properties such as symmetry and transitivity but typically required extensive training data and had limited scalability to complex relational frameworks. Although computational modeling of stimulus equivalence remains active (Tovar et al., 2023), modeling of broader AARR principles beyond stimulus equivalence is rare, with notable exceptions including recent works by Edwards et al. (2022); Edwards (2024).

In summary, relational reasoning remains a vibrant area within AI research, yet the challenge of dynamically learning arbitrary, contextually flexible relational structures with minimal training data remains largely unmet. Our proposed NARS-based framework directly addresses this gap. To the best of our knowledge, this study is the first to conceptually demonstrate how mutual entailment, combinatorial entailment, and transformation of functions—key properties of AARR—can emerge within a unified symbolic reasoning system. This theoretical foundation sets the stage for future empirical validations and positions NARS as a promising candidate for adaptive, human-like relational reasoning.

## 4 Theoretical framework: modeling AARR with NARS

To enable the modeling of Arbitrarily Applicable Relational Responding (AARR) within OpenNARS for Applications (ONA), we introduce a novel mechanism called *acquired relations*. Currently, ONA’s reasoning is based primarily on sensorimotor contingencies; however, according to NARS theory (NAL Definition 8.1 in Wang (2013)), relational terms (*products*) can equivalently be represented as compound terms of inheritance statements. This theoretical notion has not yet been implemented in ONA, and its introduction would allow the system to derive relational statements directly from learned sensorimotor contingencies.

Within NARS theory, a learned contingency such as.

<((<A1 --> p1> &/ <B1 --> q1>) &/ ^left) =/> G>.

can yield an *acquired relation*, formally represented as.

<(A1 ∗ B1) --> (p1 ∗ q1)>.

In the notation employed here, learned sensorimotor contingencies often take the form.

<(sample ∗ red) --> (loc ∗ color)> &/

<(left ∗ blue) --> (loc ∗ color)> &/

<( {SELF} ∗ (sample ∗ left)) --> ^match> =/> G>.

Following our approach, this yields two distinct relational terms—one describing the relation between stimulus properties (colors), and another describing the relational structure of stimulus locations.

<(red ∗ blue) --> (color ∗ color)> &&

<(sample ∗ left) --> (loc ∗ loc)>

To avoid a *combinatorial explosion*, i.e., an exponential growth in derived terms and inferences, the introduction of acquired relations is carefully restricted. Specifically, new relations are generated only when procedural operations within contingencies are actively executed by the system. This targeted triggering ensures computational efficiency while maintaining functional generality.

Acquired relations can be combined with *implications*, another core element in NARS theory (see statement-level inference in Wang (2013)), allowing for generalized, context-sensitive reasoning. For example, from the acquired relations shown previously, the following implications can be derived.

<(red ∗ blue) --> (color ∗ color)> &&

<(sample ∗ left) --> (loc ∗ loc)> ==>

<(sample ∗ red) --> (loc ∗ color)> &/

<(left ∗ blue) --> (loc ∗ color)> &/

<( {SELF} ∗ (sample ∗ left)) --> ^match> =/> G>.

More generally, implications abstracted with variables take this form.

<($1 ∗ $2) --> (color ∗ color)> &&

<($3 ∗ $4) --> (loc ∗ loc)> ==>

<($3 ∗ $1) --> (loc ∗ color)> &/

<($4 ∗ $2) --> (loc ∗ color)> &/

<( {SELF} ∗ ($3 ∗ $4)) --> ^match> =/> G>.

This framework can be understood as a grounding mechanism whereby abstract relations (e.g., color-color) become anchored in concrete sensorimotor experiences. This allows NARS to dynamically transition from basic, animal-like contingency learning towards symbolic, human-like reasoning capabilities.

When multiple abstract relational templates or rules could apply during inference, NARS selects among these templates by prioritizing the rule with the highest *truth expectation* (Hammer and Lofthouse, 2020). Truth expectation in NARS is calculated as a function of frequency and confidence associated with previously derived relational implications:

$$exp\left(f,c\right)=c\times \left(f-\frac{1}{2}\right)+\frac{1}{2}$$

where frequency (f) represents the proportion of positive evidence relative to the total evidence, and confidence (c) reflects the degree of evidential support based on the total amount of evidence (Hammer and Lofthouse, 2020). Thus, inference proceeds using the relational rule with the strongest combined evidential support, reflecting the system’s accumulated relational learning experiences.

Conversely, symbolic-level relational statements can also guide sensorimotor behavior. If a relation such as (blue→yellow) is symbolically derived, it can then inform decision-making in novel situations via the implications described above, provided relevant locational relations (e.g., (sample→right) ) are established through direct interaction with the environment.

The concept of acquired relations is general and not restricted to matching-to-sample procedures. For example, functional equivalences acquired through interactions with different procedures also lead to relational derivations. Consider the following example.

<(<(left ∗ green) --> (loc ∗ color)> &/

<( {SELF} ∗ left) --> ^select>) =/> G>

100

<(<(left ∗ blue) --> (loc ∗ color)> &/

<( {SELF} ∗ left) --> ^select>) =/> G>

*// Derived functional equivalence:*

<(left ∗ green) --> (loc ∗ color)> <=>

<(left ∗ blue) --> (loc ∗ color)>

This equivalence, in turn, can support acquired relational implications.

<(green ∗ blue) --> (color ∗ color)> &&

<(left ∗ left) --> (loc ∗ loc)> ==>

<(left ∗ green) --> (loc ∗ color)> <=>

<(left ∗ blue) --> (loc ∗ color)>

*// Abstracted form:*

<($1 ∗ $2) --> (color ∗ color)> &&

<($3 ∗ $3) --> (loc ∗ loc)> ==>

<($3 ∗ $1) --> (loc ∗ color)> <=>

<($3 ∗ $2) --> (loc ∗ color)>

This flexibility aligns closely with contemporary learning psychology perspectives, which argue that any regularity—such as stimulus pairing or common roles within contingencies—can serve as a contextual cue for relational responding (De Houwer and Hughes, 2020; Hughes et al., 2016).

In the following section, we detail specific experimental paradigms designed to validate and explore the capabilities enabled by these modeling extensions.

## 5 Illustrative theoretical demonstrations

The following sections present conceptual scenarios illustrating logical derivations rather than empirical experiments. These demonstrations serve as theoretical proofs-of-concept, designed to illustrate how the proposed NARS extensions could enable Arbitrarily Applicable Relational Responding (AARR). Quantitative performance metrics (e.g., accuracy, F1-score) are not applicable in this purely theoretical context but remain important targets for future empirical evaluations.

Crucially, during all theoretical testing phases reported here, we presented only the goal-event (G! :|:) to trigger system choices. We never provided feedback or reinforcement (G. :|:) during these tests. Thus, our testing phases strictly followed standard Matching-to-Sample (MTS) procedures used in human relational research, ensuring genuine tests of generalization in the absence of feedback. Please see the Supplementary Material for details.

In alignment with standard Matching-to-Sample procedures used in the human studies we replicate, the spatial positions (left/right) of comparison stimuli were systematically varied and balanced across trials within each training and testing block. This procedure, which has also been employed consistently in our previous experimental research with NARS-based systems (Johansson et al., 2023; Johansson, 2024b), ensures that relational responding could not rely on positional cues.

During training phases, we propose providing feedback in the form of positive reinforcement (G. :|:) for correct responses and negative feedback (G. :|: {0.0 0.9 }) for incorrect responses. In this conceptual framework, negative feedback would reduce the truth expectation of corresponding implications, theoretically decreasing the probability that NARS would repeat incorrect behavior. This approach allows NARS, at a theoretical level, to adapt relational knowledge based on experience. However, empirical testing of this mechanism remains an essential direction for future research.

**FIGURE 2:**
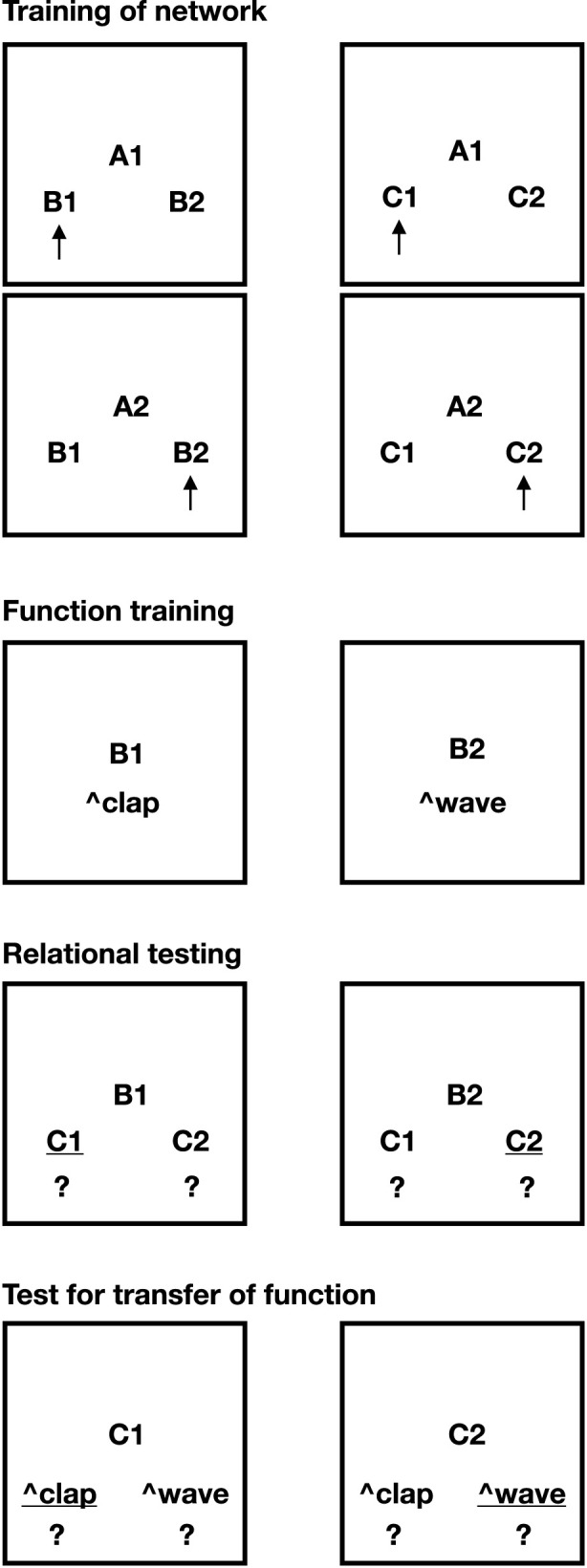
Task 1 of this paper. Stimulus equivalence and the transfer of function. The necessary pre-training (Phase 1) is excluded from the picture. Picture shows Phases 2–5 of the task. Underlined options indicate correct choices.

**FIGURE 3:**
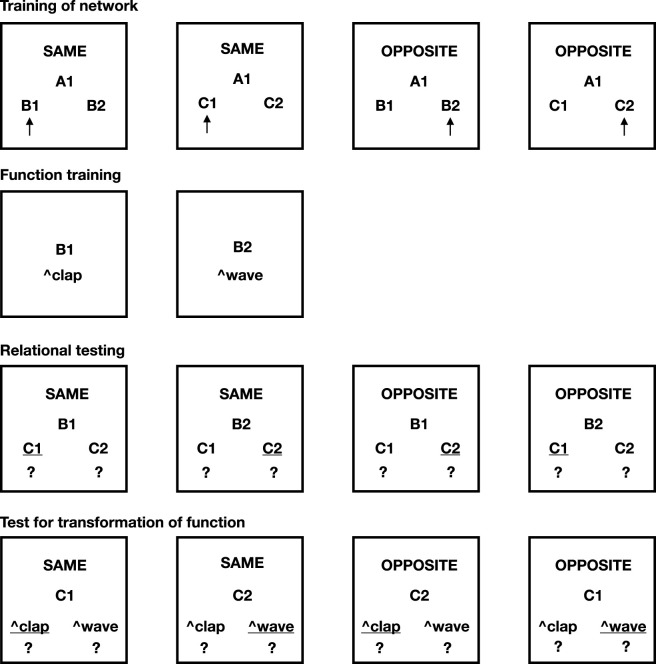
Task 2 of this paper. AARR in accordance with opposition and the transformation of function. The necessary pre-training (Phase 1) is excluded from the picture. Picture shows Phases 2–5 of the task. Underlined options indicate correct choices.

We adapted two paradigms from Relational Frame Theory (RFT) literature: the Stimulus Equivalence and Function Transfer task (Task 1; Figure 2) and the Opposition and Function Transformation task (Task 2; Figure 3) (Hayes et al., 1987; Roche et al., 2000). These tasks were modified to conceptually fit the capabilities of NARS. Importantly, these setups were not implemented empirically in OpenNARS for Applications (ONA) (Hammer and Lofthouse, 2020); rather, they are presented here as symbolic analyses intended to illustrate how NARS, when theoretically extended, could account for these forms of relational reasoning.

### 5.1 Task 1: stimulus equivalence and transfer of function

The design for Task 1 was inspired by the methodology introduced by Hayes et al. (1987). In their original human study, participants underwent four phases: (1) training conditional discriminations, (2) testing for derived equivalence classes, (3) training discriminative stimulus functions on selected class members, and (4) testing whether discriminative functions transferred to other members of the same equivalence classes. Importantly, the original study did not account for participants’ prior relational learning history.

In the present study, we included pretraining to establish basic relational skills prior to the main experiments. The study consisted of four phases conducted sequentially.

1. Pretraining of relational networks: This phase explicitly trained foundational relations such as symmetry ( X1→Y1 and Y1→X1 ), and transitivity ( X1→Y1 , Y1→Z1 , thus deriving X1→Z1 ).
2. Training conditional discriminations: Using a Matching-to-sample (MTS) procedure, conditional discriminations were trained within two separate stimulus networks: one comprising stimuli A1 , B1 , and C1 , and another comprising A2 , B2 , and C2 .
3. Function training: NARS was trained to execute two discriminative responses: ^clap when B1 was presented as a sample stimulus, and ^wave when B2 appeared as the sample.
4. Testing derived relations and transfer: In the final phase, derived relations within each ABC network were tested without feedback, specifically examining whether previously trained discriminative functions (^clap, ^wave) transferred to equivalent stimuli ( C1 , C2 ).

### 5.2 Task 2: opposition and transformation of function

Task 2 was inspired by the relational methodology of Roche et al. (2000). Roche and colleagues examined how derived relational responses and stimulus functions transformed contextually using “Same” and “Opposite” relational frames. Their human participants initially learned operant associations between arbitrary stimuli and actions (e.g., waving, clapping), followed by relational pretraining to establish “Same” and “Opposite” frames. Through training and contextual cueing, participants showed contextually controlled derived responding (e.g., relationally responding “Same” or “Opposite” for specific stimuli) and function transformation.

In the current study, we again included explicit pretraining phases to equip NARS with necessary relational skills. The experimental design comprised five phases.

1. Pretraining of relational frames: This phase explicitly trained “SAME” and “OPPOSITE” relations, establishing mutual entailment (e.g., SAME X1↔Y1 , OPPOSITE X1↔Y2 ) and combinatorial entailment (e.g., SAME X1→Y1 , SAME Y1→Z1 , thus deriving SAME X1→Z1 ). Functional equivalence and transfers between symmetry and functional equivalence were also established.
2. Training relational networks: Using the Matching-to-sample (MTS) procedure, relational networks were trained, forming SAME (e.g., A1→B1 , A1→C1 ) and OPPOSITE ( A1→B2 , A1→C2 ) relations. A second analogous network ( A2 - B2 - C2 ) was similarly trained.
3. Function training: The system was trained to produce discriminative responses ^clap (for B1 ) and ^wave (for B2 ).
4. Testing derived relations and function transformations: In the final phase, derived relations within the SAME/OPPOSITE networks were tested without feedback, specifically examining whether trained functions transformed appropriately across relational contexts. Stimuli tested included combinations such as SAME/ C1 , SAME/ C2 , OPPOSITE/ C1 , and OPPOSITE/ C2 .

## 6 Theoretical results and conceptual derivations

Given the detailed and extensive nature of the logical derivations underlying these theoretical demonstrations, the full derivations, explicit representations, and step-by-step processes are presented in the Supplementary Material. Here, we summarize the key outcomes of our theoretical demonstrations evaluating whether NARS, with the proposed extensions, can model Arbitrarily Applicable Relational Responding (AARR). The main text thus maintains readability by focusing on the key relational properties (mutual entailment, combinatorial entailment, and transformation of function) that conceptually emerge within the NARS framework.

### 6.1 Stimulus equivalence and transfer of function

In the first experiment (illustrated in Figure 2), we explored whether NARS logic could model the formation of stimulus equivalence classes and demonstrate the transfer of stimulus functions across related stimuli. Briefly, NARS was theoretically exposed to matching-to-sample (MTS) procedures where conditional relations ( A→B and B→C ) were trained. Additionally, discriminative functions were assigned to specific stimuli within these relational networks (e.g., stimulus $B_{1}$ triggering a ^clap response, and $B_{2}$ a ^wave response).

Key results included.

- Mutual entailment: NARS successfully derived bidirectional relations (e.g., if trained A→B , it inferred B→A ).
- Combinatorial entailment: The system correctly inferred indirect relations from explicitly trained ones (e.g., from A→B and B→C , it inferred A→C ).
- Transformation of function: Critically, discriminative functions (e.g., ^clap and ^wave) initially trained on B -stimuli were transferred without additional training to C -stimuli through derived equivalence relations, demonstrating a successful relational transfer of stimulus functions.

**FIGURE 4:**
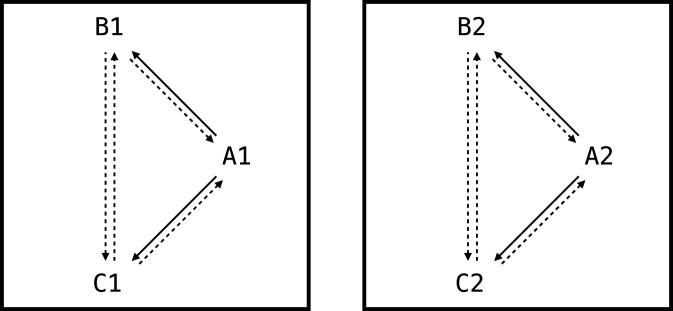
The two networks trained as part of the first experiment of this paper. Solid arrows represent relations that are explicitly trained. Dashed arrows represent derived relations.

Thus, NARS logic adequately models essential aspects of stimulus equivalence and function transfer, foundational within Relational Frame Theory (Figure 4; detailed derivations in Supplementary Material Section S1).

### 6.2 Opposition and transformation of function

In the second experiment (illustrated in Figure 3), we assessed whether NARS logic could model relational networks involving oppositional frames (“SAME” and “OPPOSITE”) and the contextual transformation of stimulus functions. Similar to the first task, MTS training was theoretically applied, but now relations involved both SAME and OPPOSITE contexts. After training, discriminative functions were again assigned to specific stimuli within these networks.

Key outcomes included.

- Context-sensitive mutual entailment and combinatorial entailment: NARS derived relations consistent with trained SAME and OPPOSITE relational frames, correctly generalizing from trained examples.
- Transformation of function across oppositional relations: Trained discriminative functions (e.g., ^clap associated with stimulus $B_{1}$ , and ^wave with $B_{2}$ ) were accurately transferred to related stimuli ( $C_{1}$ and $C_{2}$ ), including appropriate reversal in functions when oppositional relational contexts were applied (e.g., if stimulus pairs were related as OPPOSITE, stimulus functions reversed accordingly).

**FIGURE 5:**
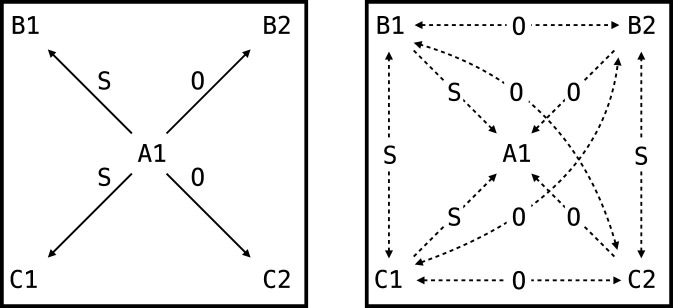
The network trained as part of the second experiment of this paper. S and O indicate SAME and OPPOSITE, respectively. Left panel shows relations that are explicitly trained. Right panel shows derived relations.

These results illustrate that NARS logic effectively models complex, contextually controlled transformations of function, consistent with Relational Frame Theory (Figure 5; detailed derivations in Supplementary Material Section S2).

In summary, these theoretical demonstrations confirm that the extended NARS logic is sufficiently powerful and flexible to capture core relational learning phenomena—mutual entailment, combinatorial entailment, and transformation of function—essential for modeling human-like symbolic reasoning and cognition.

## 7 Discussion

This study demonstrated that the Non-Axiomatic Reasoning System (NARS), extended with mechanisms inspired by Relational Frame Theory (RFT), can successfully model Arbitrarily Applicable Relational Responding (AARR), a cornerstone of human cognition. Through theoretical analysis and logical derivations, we showed how NARS’s adaptive logic can capture essential relational learning phenomena without pre-defined axioms or extensive data-driven training. This integration provides a computational framework aligning cognitive science principles with artificial intelligence (AI), underscoring the interdisciplinary potential of Machine Psychology (Johansson, 2024a; Johansson, 2024b) in developing flexible, context-sensitive reasoning systems.

### 7.1 Summary of theoretical insights

We have shown theoretically that NARS can replicate critical aspects of human-like relational reasoning by modeling Arbitrarily Applicable Relational Responding. Specifically, we demonstrated that.

NARS exhibits *mutual entailment*, accurately deriving bidirectional relations from trained unidirectional associations.
It demonstrates robust *combinatorial entailment*, integrating multiple trained relations to correctly infer novel relations.
It successfully replicates *transformation of stimulus function*, whereby functions (such as specific responses like “clap” or “wave”) trained to one stimulus are systematically transferred to other related stimuli without additional direct training.

These findings illustrate that the cognitive mechanisms underlying AARR—once considered unique to biologically evolved cognition—can be conceptually instantiated within a symbolic reasoning system. NARS’s capability to learn from minimal, structured experiences and subsequently perform flexible relational inference provides a clear departure from contemporary AI models that primarily rely on large-scale statistical training. Instead, our approach emphasizes “small data” and logical consistency, aligning closely with the RFT premise that very few exemplars, combined with appropriate contextual cues, can generate powerful relational generalizations.

### 7.2 Implications for artificial general intelligence

Our theoretical demonstration of AARR within NARS offers significant implications for AGI research. First, it illustrates that sophisticated relational reasoning is achievable through adaptive symbolic systems without relying on extensive datasets, reinforcing structured symbolic learning as a viable path toward AGI. Second, our approach establishes learning psychology principles—particularly those articulated by RFT—as functional benchmarks for evaluating AGI systems’ relational generalization capabilities. Third, the flexibility of NARS in dynamically constructing relational structures under uncertainty makes it suitable for adaptive, real-world contexts. Lastly, integrating adaptive logic with relational reasoning supports broad applications, including robotics and human-AI interaction, where context-sensitive symbolic manipulation is essential for achieving human-like understanding.

### 7.3 Limitations and future research directions

This theoretical study presents a conceptual framework and logical derivations rather than empirical validation. As such, the proposed extensions to NARS have not yet been practically implemented or empirically tested within an actual NARS-based AI system. Quantitative evaluations, such as measuring accuracy, precision, recall, or F1-score of learned relational structures, are therefore not presented in this study. Empirical validation—including quantitative performance assessments and comparative baseline evaluations with established methods such as Inductive Logic Programming (ILP), Statistical Relational Learning (SRL), and Neural Logic Machines—remains essential future work.

Furthermore, our theoretical demonstrations employed binary (two-choice) comparisons rather than multi-choice comparison tasks typically found in human MTS studies, thereby simplifying the generalization and discrimination demands. Future empirical validations should implement multi-choice comparison setups to systematically assess the scalability and generalization of relational responding within the NARS framework.

Several other avenues remain open for further exploration. One immediate direction involves expanding the relational frames modeled in NARS beyond equivalence and opposition, including comparative, hierarchical, and deictic relations, to comprehensively evaluate the system’s generalization capabilities. Another promising direction involves scaling relational networks by increasing stimulus complexity, testing NARS’s resource management and inference flexibility. Additionally, integrating perceptual inputs with symbolic reasoning represents a crucial step toward practical, embodied applications, enabling NARS to generate and reason about relations directly from sensory data in dynamic environments. Lastly, further refining and automating the relational learning mechanisms within NARS, alongside comparisons of NARS-derived relational learning curves with empirical human data, could guide targeted enhancements and deepen our understanding of relational cognition in both artificial and biological systems.

## 8 Conclusion

We presented a theoretical framework demonstrating that NARS, enhanced by relational learning principles derived from Relational Frame Theory, can successfully model Arbitrarily Applicable Relational Responding—a foundational component of human cognition. This provides a concrete method for developing symbolic AI systems capable of dynamic, context-sensitive relational reasoning similar to that observed in humans. These findings represent a meaningful step toward bridging cognitive science and artificial intelligence, emphasizing that principles identified through human learning research can inform AI systems that “think” more like humans—not necessarily in brain-like structures but in the dynamic and contextually controlled use of symbolic knowledge. Continued interdisciplinary research in this direction holds considerable promise for developing flexible, adaptive, and ultimately more human-like artificial intelligence.

## Data Availability

The raw data supporting the conclusions of this article will be made available by the authors, without undue reservation.
