# Supplementary material for: Modeling arbitrarily applicable relational responding with the non-axiomatic reasoning system: a Machine Psychology approach
Source: Front Robot AI. 2025 Sep 22;12:1586033. doi: 10.3389/frobt.2025.1586033 (PMC12497618; doi:10.3389/frobt.2025.1586033)
Supplement: Supplementary file 1 [file DataSheet1.pdf]

Supplementary Material

CLARIFICATION OF NARSESE SYNTAX AND KEY CONCEPTS

To enhance readability and reproducibility, we provide clear definitions and explanations of essential concepts and symbols used in the NARS framework:

- **Motor Babbling:** A process where the NARS system randomly executes operations (actions) when it lacks sufficient learned procedural knowledge. Motor babbling allows the system to explore different actions, enabling the formation of initial sensorimotor contingencies (Johansson, 2024).
- **Goal and Feedback Symbols:**
  - `G! :|:` establishes "G" as a goal event, prompting the NARS system to execute a decision or action.
  - `G. :|:` indicates that "G" is provided as feedback or reinforcement following the execution of an action. Positive feedback is represented as `G. :|:`, while negative feedback (indicating an incorrect choice) is represented explicitly as `G. :|: {0.0 0.9}` (Johansson, 2024).
- **Frequency and Confidence:** Truth values in NARS are represented using two measures—frequency (f) and confidence (c), defined as:

  $$f = \frac{w^+}{w}, \quad c = \frac{w}{w+1}$$

  where w^+ is the positive evidence, and w is the total evidence (positive and negative combined). Frequency represents the proportion of positive evidence, while confidence reflects the amount of total evidence collected. Both values range between 0 and 1, where a frequency of 1.0 indicates that all accumulated evidence has been positive, and higher confidence values signify greater evidential support (Hammer and Lofthouse, 2020; Hammer, 2022; Johansson, 2024).
- **Timing Conventions (time steps):** Time steps represent temporal spacing between events and actions in NARS. A notation such as `100` indicates that 100 discrete temporal steps have passed between two events. Temporal spacing influences inference, memory decay, and the updating of truth values (frequency and confidence). Larger time intervals between events generally result in decreased influence of those events on truth values and predictions (Johansson, 2024).

During the testing phases described in this Supplementary Material, only the goal event (`G! :|:`) was presented to trigger the system's choice. No feedback or reinforcement (`G. :|:`) was provided, ensuring that the testing procedures remained feedback-free and consistent with standard generalization testing in Matching-to-Sample (MTS) studies.

1 STIMULUS EQUIVALENCE AND FUNCTION TRANSFER TASK

1.1 Results

1.1.1 Phase 1: Pretraining

1.1.1.1 *Learning Conditionality*

First, the $X1 \rightarrow Y1$ relation was trained using the Matching-to-sample procedure:

```
<(sample * X1) --> (loc * ocr)>. :|:
<(left * Y1) --> (loc * ocr)>. :|:
<(right * Y2) --> (loc * ocr)>. :|:
G! :|: // Establish G as a goal
```

Motor babbling would be triggered, and with feedback, `G` would be provided as a consequence when `<({SELF} * (sample * left)) --> ^match>` was executed.

After execution, the following contingency would be derived:

```
<(sample * X1) --> (loc * ocr)> &/ <(left * Y1) --> (loc * ocr)> &/
   <({SELF} * (sample * left)) --> ^match>) =/> G>.
```

Since the system acted on this contingency, the following two relations would be acquired:

```
<(X1 * Y1) --> (ocr * ocr)> && <(sample * left) --> (loc * loc)>.
```

With these derived, an implication between the acquired $X1 \rightarrow Y1$ and $sample \rightarrow left$ relations and the corresponding contingency would also be derived:

```
<(X1 * Y1) --> (ocr * ocr)> && <(sample * left) --> (loc * loc)>
 ==>
<(sample * X1) --> (loc * ocr)> &/ <(left * Y1) --> (loc * ocr)>
 &/ <({SELF} * (sample * left)) --> ^match>) =/> G>
```

Finally, a general form of this implication, with variables introduced, would also be established:

```
<($1 * $2) --> (ocr * ocr)> && <($3 * $4) --> (loc * loc)>
 ==>
<($3 * $1) --> (loc * ocr)> &/ <($4 * $2) --> (loc * ocr)> &/
 <({SELF} * ($3 * $4)) --> ^match>) =/> G>
```

1.1.1.2 *Learning Symmetry*

After the $X1 \rightarrow Y1$ relation has been trained, the system could be exposed to a matching-to-sample situation where the $Y1 \rightarrow X1$ could be trained:

```
<(sample * Y1) --> (loc * ocr)>. :|:
<(left * X1) --> (loc * ocr)>. :|:
<(right * X2) --> (loc * ocr)>. :|:
G! :|:
```

With similar learning as in the last paragraph, the system would acquire the following:

```
<(Y1 * X1) --> (ocr * ocr)> && <(sample * left) --> (loc * loc)>
 ==>
<(sample * Y1) --> (loc * ocr)> &/ <(left * X1) --> (loc * ocr)>
 &/ <({SELF} * (sample * left)) --> ^match>) =/> G>
```

Symmetry, represented with a functional equivalence, could then be derived using the acquired $X1 \rightarrow Y1$ and $Y1 \rightarrow X1$ relations.

```
<(X1 * Y1) --> (ocr * ocr)> <=> <(Y1 * X1) --> (ocr * ocr)>

<($1 * $2) --> (ocr * ocr)> <=> <($2 * $1) --> (ocr * ocr)>
```

1.1.1.3 Learning Transitivity

With experience of the $X1 \rightarrow Y1$, $Y1 \rightarrow Z1$ and $X1 \rightarrow Z1$ relations (in the matching-to-sample), the system will be trained explicitly on transitivity. The following will be derived:

```
<(X1 * Y1) --> (ocr * ocr)> && <(Y1 * Z1) --> (ocr * ocr)> ==>
 <(X1 * Z1) --> (ocr * ocr)>

<($1 * $2) --> (ocr * ocr)> && <($2 * $3) --> (ocr * ocr)> ==>
 <($1 * $3) --> (ocr * ocr)>
```

1.1.1.4 Learning a Relation From Functional Equivalence

As described in the main paper, the idea of acquiring relations can be generalized to any procedure. Here it is assumed to be possible also in a functional equivalence procedure.

First, two three-term contingencies are learned, where $X1$ and $Y1$ both function as a discriminative stimulus.

```
<(sample * X1) --> (loc * ocr)>. :|:
<({SELF} * op1) --> ^action>. :|:
G. :|: // Provide G as a consequence

100 // Wait 100 time steps

<(sample * Y1) --> (loc * ocr)>. :|:
<({SELF} * op1) --> ^action>. :|:
G. :|:
```

A functional equivalence between the two $X1$ and $Y1$ events that had the same functional role (discriminative stimulus) would be obtained:

```
<(sample * X1) --> (loc * ocr)> <=>
 <(sample * Y1) --> (loc * ocr)>
```

Importantly, the system could also acquire a relation of this kind:

```
<(X1 * Y1) --> (ocr * ocr)> &&
 <(sample * sample) --> (loc * loc)>
```

```
 ==>
 <(sample * X1) --> (loc * ocr)> <=>
  <(sample * Y1) --> (loc * ocr)>
```

An abstract version of this would also be derived:

```
<($1 * $2) --> (ocr * ocr)> && <($3 * $3) --> (loc * loc)> ==>
 <($3 * $1) --> (loc * ocr)> <=>
 <($3 * $2) --> (loc * ocr)>
```

1.1.2 Phase 2: Training of Networks

The ABC networks (Figure 4) were trained using the matching-to-sample procedure. For example, the $A1 \rightarrow B1$ could be trained using feedback as follows:

```
<(sample * A1) --> (loc * ocr)>. :|:
<(left * B1) --> (loc * ocr)>. :|:
<(right * B2) --> (loc * ocr)>. :|:
// Executed with for example motor babbling
<({SELF} * (sample * left)) --> ^match>. :|:
G. :|:

<(sample * A1) --> (loc * ocr)> &/ <(left * B1) --> (loc * ocr)>
 &/ <({SELF} * (sample * left)) --> ^match>) =/> G>

<(A1 * B1) --> (ocr * ocr)> && <(sample * left) --> (loc * loc)>
 ==>
<(sample * A1) --> (loc * ocr)> &/ <(left * B1) --> (loc * ocr)>
 &/ <({SELF} * (sample * left)) --> ^match>) =/> G>
```

In all, the $A1 - B1 - C1$ network (Figure 4) with the derivations would be as follows:

```
// (1) Trained
<(A1 * B1) --> (ocr * ocr)>

// (2) Trained
<(A1 * C1) --> (ocr * ocr)>

// (3) Derived by symmetry from (1)
<(B1 * A1) --> (ocr * ocr)>

// (4) Derived by symmetry from (2)
<(C1 * A1) --> (ocr * ocr)>

// (5) Derived by transitivity from (3)+(2)
<(B1 * C1) --> (ocr * ocr)>

// (6) Derived by symmetry from (5)
```

```
<(C1 * B1) --> (ocr * ocr)>
```

The corresponding $A2 - B2 - C2$ network (Figure 4) would also be derived.

1.1.3 Phase 3: Function Training

In this phase, two discriminative functions were trained for $B1$ and $B2$, respectively.

```
<(sample * B1) --> (loc * ocr)>. :|:

// Babbled or explicitly shown to the system
<({SELF} * clap) --> ^action>. :|:
G. :|:

// Derived:
<(sample * B1) --> (loc * ocr)>) &/
 <({SELF} * clap) --> ^action> =/> G>.

<(sample * B2) --> (loc * ocr)>. :|:
<({SELF} * wave) --> ^action>. :|:
G. :|:

// Derived:
<(sample * B2) --> (loc * ocr)>) &/
 <({SELF} * wave) --> ^action> =/> G>.
```

1.1.4 Phase 4: Relational Testing

The first part of the testing, concerning matching-to-sample, would involve a situation like the following.

```
<(sample * C1) --> (loc * ocr)>. :|:
<(left * B1) --> (loc * ocr)>. :|:
<(right * B2) --> (loc * ocr)>. :|:
G! :|:
```

First, by looking at / interacting with the scene, the system is assumed to acquire the following relation:

```
<(sample * left) --> (loc * loc)>
```

The following relation has been derived from before:

```
<(C1 * B1) --> (ocr * ocr)>
```

With the general form of conditionality from Paragraph 1.1.1.1, then the following could be derived:

```
<(C1 * B1) --> (ocr * ocr)> && <(sample * left) --> (loc * loc)>
 ==>
<(sample * C1) --> (loc * ocr)> &/ <(left * B1) --> (loc * ocr)>) &/
   <({SELF} * (sample * left)) --> ^match>) =/> G>.
```

Hence, the operation `<({SELF} * (sample * left)) --> ^match>` will be executed.

1.1.5 Phase 5: Test for Transfer of Function

In the second part of testing, the following situation was presented to the system:

```
<(sample * C1) --> (loc * ocr)>. :|:
G! :|:
```

By looking at the scene, the system is assumed to be able to acquire the following relation:

```
<(sample * sample) --> (loc * loc)>
```

With the derived parts of the network (including $C1 \rightarrow B1$) and the abstract form learned in 1.1.1.4, then the following would be derived:

```
<(C1 * B1) --> (ocr * ocr)> &&
 <(sample * sample) --> (loc * loc)> ==>
<(sample * C1) --> (loc * ocr)> <=>
<(sample * B1) --> (loc * ocr)>
```

then the operation `<({SELF} * clap) --> ^action>` would be executed, by substituting

```
<(sample * C1) --> (loc * ocr)>. :|:
```

for

```
<(sample * B1) --> (loc * ocr)>. :|:
```

and using the previously trained contingency:

```
<(sample * B1) --> (loc * ocr)>) &/ <({SELF} * clap) --> ^action>)
   =/> G>
```

Similarly, the following situation

```
<(sample * C2) --> (loc * ocr)>. :|:
G! :|:
```

would lead to the operation `<({SELF} * wave) --> ^action>` being executed, using similar derivations.

2 OPPOSITION AND FUNCTION TRANSFORMATION TASK

2.1 Results

2.1.1 Phase 1: Pretraining of All Relations

2.1.1.1 Learning the Acquired Relations

In the example below, `rel` is also used as a location, as illustrated in Figure 1 from an earlier chapter. Initial training of the relation form would be trained using the matching-to-sample procedure:

```
<(rel * SAME) --> (loc * ocr)>. :|:
<(sample * X1) --> (loc * ocr)>. :|:
<(left * Y1) --> (loc * ocr)>. :|:
```

```
<(right * Y2) --> (loc * ocr)>. :|:
G! :|:
```

When learning (with feedback) to match the sample and the left option, the following contingency would be formed:

```
<(rel * SAME) --> (loc * ocr)> &/
 <(sample * X1) --> (loc * ocr)> &/
 <(left * Y1) --> (loc * ocr)> &/
 <({SELF} * (sample * left)) --> ^match>) =/> G>
```

An acquired relation SAME $X1 \rightarrow Y1$ (and a $(rel \times (sample \times left))$ relation) with corresponding implication will be formed:

```
<(SAME * (X1 * Y1)) --> (ocr * (ocr * ocr))> &&
 <(rel * (sample * left)) --> (loc * (loc * loc))>
 ==>
 <(rel * SAME) --> (loc * ocr)> &/
  <(sample * X1) --> (loc * ocr)> &/
  <(left * Y1) --> (loc * ocr)> &/
  <({SELF} * (sample * left)) --> ^match>) =/> G>
```

The abstract version that is also derived, with variables introduced, would be the following:

```
<($1 * ($2 * $3)) --> (ocr * (ocr * ocr))> &&
 <($4 * ($5 * $6)) --> (loc * (loc * loc))>
 ==>
 <($4 * $1) --> (loc * ocr)> &/
  <($5 * $2) --> (loc * ocr)> &/
  <($6 * $3) --> (loc * ocr)> &/
  <({SELF} * ($5 * $6)) --> ^match>) =/> G>
```

2.1.1.2 Learning Mutual Entailment of SAME and OPPOSITE

The system will be explicitly trained on the SAME $X1 \rightarrow Y1$ and SAME $Y1 \rightarrow X1$ relations, using the matching-to-sample.

After training, mutual entailment for the SAME relation would be formed. First:

```
<(SAME * (X1 * Y1)) --> (ocr * (ocr * ocr))> &&
 <(rel * (sample * left)) --> (loc * (loc * loc))>
 <=>
<(SAME * (Y1 * X1)) --> (ocr * (ocr * ocr))> &&
 <(rel * (sample * left)) --> (loc * (loc * loc))>
```

This would be further reduced to:

```
<(SAME * (X1 * Y1)) --> (ocr * (ocr * ocr))>
 <=>
<(SAME * (Y1 * X1)) --> (ocr * (ocr * ocr))>
```

The abstract form would be:

```
<($1 * ($2 * $3)) --> (ocr * (ocr * ocr))>
 <=>
<($1 * ($3 * $2)) --> (ocr * (ocr * ocr))>
```

For OPPOSITE $X1 \rightarrow Y2$ and OPPOSITE $Y2 \rightarrow X1$ the representation of mutual entailment would be the same. It is only at the level of combinatorial entailment (see next paragraph) where these patterns differ.

2.1.1.3 Learning Combinatorial Entailment

The following four concrete and four abstract patterns of combinatorial entailment would be explicitly trained.

For SAME-SAME combinations:

```
<(SAME * (X1 * Y1)) --> (ocr * (ocr * ocr))> &&
 <(SAME * (Y1 * Z1)) --> (ocr * (ocr * ocr))> ==>
  <(SAME * (X1 * Z1)) --> (ocr * (ocr * ocr))>

<($1 * ($2 * $3)) --> (ocr * (ocr * ocr))> &&
 <($1 * ($3 * $4)) --> (ocr * (ocr * ocr))> ==>
  <($1 * ($2 * $4)) --> (ocr * (ocr * ocr))>
```

For SAME-OPPOSITE combinations:

```
<(SAME * (X1 * Y1)) --> (ocr * (ocr * ocr))> &&
 <(OPPOSITE * (Y1 * Z2)) --> (ocr * (ocr * ocr))> ==>
  <(OPPOSITE * (X1 * Z2)) --> (ocr * (ocr * ocr))>

<(SAME * ($1 * $2)) --> (ocr * (ocr * ocr))> &&
 <($3 * ($2 * $4)) --> (ocr * (ocr * ocr))> ==>
  <($3 * ($1 * $4)) --> (ocr * (ocr * ocr))>
```

For OPPOSITE-SAME combinations:

```
<(OPPOSITE * (X1 * Y2)) --> (ocr * (ocr * ocr))> &&
 <(SAME * (Y2 * Z2)) --> (ocr * (ocr * ocr))> ==>
  <(OPPOSITE * (X1 * Z2)) --> (ocr * (ocr * ocr))>

<($1 * ($2 * $3)) --> (ocr * (ocr * ocr))> &&
 <(SAME * ($3 * $4)) --> (ocr * (ocr * ocr))> ==>
  <($1 * ($2 * $4)) --> (ocr * (ocr * ocr))>
```

For OPPOSITE-OPPOSITE combinations:

```
<(OPPOSITE * (X1 * Y2)) --> (ocr * (ocr * ocr))> &&
 <(OPPOSITE * (Y2 * Z1)) --> (ocr * (ocr * ocr))> ==>
  <(SAME * (X1 * Z1)) --> (ocr * (ocr * ocr))>

<($1 * ($2 * $3)) --> (ocr * (ocr * ocr))> &&
```

```
<($1 * ($3 * $4)) --> (ocr * (ocr * ocr))> ==>
  <(SAME * ($2 * $4)) --> (ocr * (ocr * ocr))>
```

2.1.1.4 Learning a Relation From Functional Equivalence

For the system to be able to generalize from the Matching-to-sample procedure to a network of discriminative functions, the following was presented as part of the pretraining.

```
<(rel * SAME) --> (loc * ocr)>. :|:
<(sample * X1) --> (loc * ocr)>. :|:
<({SELF} * op1) --> ^action>. :|:
G. :|:

100 // Wait 100 time steps

<(sample * Y1) --> (loc * ocr)>. :|:
<({SELF} * op1) --> ^action>. :|:
G. :|:
```

A functional equivalence between the preconditions that functioned as discriminative stimuli would be obtained:

```
<(rel * SAME) --> (loc * ocr)> &/ <(sample * X1) --> (loc * ocr)>
 <=>
<(sample * Y1) --> (loc * ocr)>
```

Second, an acquired relation would be derived, with the corresponding implication:

```
<(SAME * (X1 * Y1)) --> (ocr * (ocr * ocr))> &&
 <(rel * (sample * sample)) --> (loc * (loc * loc))>
 ==>
 <(rel * SAME) --> (loc * ocr)> &/
  <(sample * X1) --> (loc * ocr)>
  <=>
 <(sample * Y1) --> (loc * ocr)>
```

The abstract form would be the following:

```
<($1 * ($2 * $3)) --> (ocr * (ocr * ocr))> &&
 <($4 * ($5 * $5)) --> (loc * (loc * loc))>
 ==>
 <($4 * $1) --> (loc * ocr)> &/ <($5 * $2) --> (loc * ocr)>
  <=>
 <($5 * $3) --> (loc * ocr)>
```

Regarding learning the OPPOSITE relation with discriminative functions, the following was presented.

```
<(rel * OPPOSITE) --> (loc * ocr)>. :|:
<(sample * X1) --> (loc * ocr)>. :|:
<({SELF} * op2) --> ^action>. :|:
```

```
G. :|:

100 // Wait 100 time steps

<(sample * Y2) --> (loc * ocr)>. :|:
<({SELF} * op2) --> ^action>. :|:
G. :|:
```

A functional equivalence would be obtained:

```
<(rel * OPPOSITE) --> (loc * ocr)> &/ <(sample * X1) --> (loc * ocr)>
 <=>
<(sample * Y2) --> (loc * ocr)>
```

Then, an acquired relation would be derived, with a corresponding implication:

```
<(OPPOSITE * (X1 * Y2)) --> (ocr * (ocr * ocr))> &&
 <(rel * (sample * sample)) --> (loc * (loc * loc))>
 ==>
 <(rel * OPPOSITE) --> (loc * ocr)> &/ <(sample * X1) --> (loc * ocr)
    >
  <=>
 <(sample * Y2) --> (loc * ocr)>
```

The abstract form would be:

```
<($1 * ($2 * $3)) --> (ocr * (ocr * ocr))> &&
 <($4 * ($5 * $5)) --> (loc * (loc * loc))>
 ==>
 <($4 * $1) --> (loc * ocr)> &/ <($5 * $2) --> (loc * ocr)>
  <=>
 <($5 * $3) --> (loc * ocr)>
```

Importantly, this is the same abstract form as for that of the SAME relation.

2.1.2 Phase 2: Training of Network

In this phase, it will be illustrated how the network (as illustrated in Figure 5) will be trained using the Matching-to-sample procedure, with SAME and OPPOSITE cues:

```
<(rel * SAME) --> (loc * ocr)>. :|:
<(sample * A1) --> (loc * ocr)>. :|:
<(left * B1) --> (loc * ocr)>. :|:
<(right * B2) --> (loc * ocr)>. :|:
G! :|:
```

After motor babbling, and feedback, the system would be trained into the following:

```
<(SAME * (A1 * B1)) --> (ocr * (ocr * ocr))> &&
 <(rel * (sample * left)) --> (loc * (loc * loc))>
```

```
 ==>
 <(rel * SAME) --> (loc * ocr)> &/
  <(sample * A1) --> (loc * ocr)> &/
  <(left * B1) --> (loc * ocr)> &/
  <({SELF} * (sample * left)) --> ^match>) =/> G>
```

After complete training of the entire relational network, the system would have been trained in, and having derived the following relations using the mutual and combinatorial entailment derived in Paragraphs 2.1.1.2 and 2.1.1.3:

```
// (1) Trained
<(SAME * (A1 * B1)) --> (ocr * (ocr * ocr))>

// (2) Trained
<(SAME * (A1 * C1)) --> (ocr * (ocr * ocr))>

// (3) Trained
<(OPPOSITE * (A1 * B2)) --> (ocr * (ocr * ocr))>

 // (4) Trained
<(OPPOSITE * (A1 * C2)) --> (ocr * (ocr * ocr))>

 // (5) Derived by mutual entailment from (1)
<(SAME * (B1 * A1)) --> (ocr * (ocr * ocr))>

// (6) Derived by mutual entailment from (2)
<(SAME * (C1 * A1)) --> (ocr * (ocr * ocr))>

// (7) Derived by combinatorial entailment from (5)+(2)
<(SAME * (B1 * C1)) --> (ocr * (ocr * ocr))>

// (8) Derived by mutual entailment from (7)
<(SAME * (C1 * B1)) --> (ocr * (ocr * ocr))>

// (9) Derived by combinatorial entailment from (6)+(3)
<(OPPOSITE * (C1 * B2)) --> (ocr * (ocr * ocr))>

// (10) Derived by mutual entailment from (3)
<(OPPOSITE * (B2 * A1)) --> (ocr * (ocr * ocr))>

// (11) Derived by mutual entailment from (4)
<(OPPOSITE * (C2 * A1)) --> (ocr * (ocr * ocr))>

// (12) Derived by combinatorial entailment from (11)+(1)
<(OPPOSITE * (C2 * B1)) --> (ocr * (ocr * ocr))>
```

```
// (13) Derived by combinatorial entailment from (10)+(4)
<(SAME * (B2 * C2)) --> (ocr * (ocr * ocr))>

// (14) Derived by mutual entailment from (13)
<(SAME * (C2 * B2)) --> (ocr * (ocr * ocr))>
```

2.1.3 Phase 3: Function Training

In this phase, two discriminative functions were trained for $B1$ and $B2$, respectively.

```
<(sample * B1) --> (loc * ocr)>. :|:
<({SELF} * clap) --> ^action>. :|:
G. :|:

// Derived:
<(sample * B1) --> (loc * ocr)> &/ <({SELF} * clap) --> ^action> =/>
   G>.

<(sample * B2) --> (loc * ocr)>. :|:
<({SELF} * wave) --> ^action>. :|:
G. :|:

// Derived:
<(sample * B2) --> (loc * ocr)> &/ <({SELF} * wave) --> ^action> =/>
   G>.
```

2.1.4 Phase 4: Relational Testing

The first part of the testing would involve a situation like the following, where for example the derived relations SAME $C1 \rightarrow B1$ and OPPOSITE $C1 \rightarrow B1$ would be tested.

```
<(rel * SAME) --> (loc * ocr)>. :|:
<(sample * C1) --> (loc * ocr)>. :|:
<(left * B1) --> (loc * ocr)>. :|:
<(right * B2) --> (loc * ocr)>. :|:
G! :|:
```

First, by looking at the scene, it is assumed that the system would acquire the following relation:

```
<(rel * (sample * left)) --> (loc * (loc * loc))>
```

The following relation has been derived from before:

```
<(SAME * (C1 * B1)) --> (ocr * (ocr * ocr))>
```

With the abstract form from Paragraph 2.1.1.1, then the following could be derived:

```
<(SAME * (C1 * B1)) --> (ocr * (ocr * ocr))> && <(rel * (sample *
   left)) --> (loc * (loc * loc))>
 ==>
```

```
<(rel * SAME) --> (loc * ocr)> &/ <(sample * C1) --> (loc * ocr)> &/
   <(left * B1) --> (loc * ocr)>) &/ <({SELF} * (sample * left)) --> ^
   match>) =/> G>.
```

Hence, the operation `<({SELF} * (sample * left)) --> ^match>` would be executed.

2.1.5 Phase 5: Test for Transformation of Function

In the final phase, the following situation was presented to the system (Bottom row of Figure 3:

```
<(rel * SAME) --> (loc * ocr)>. :|:
<(sample * C1) --> (loc * ocr)>. :|:
G! :|:
```

Assuming that the system, by interacting with the situation would acquire:

```
<(rel * (sample * sample)) --> (loc * (loc * loc))>
```

The system would also use the following previously derived relation:

```
<(SAME * (C1 * B1)) --> (ocr * (ocr * ocr))>
```

Those two relations would derive the following functional equivalence, using the abstract form for SAME as described in Paragraph 2.1.1.4:

```
<(SAME * (C1 * B1)) --> (ocr * (ocr * ocr))> &&
 <(rel * (sample * sample)) --> (loc * (loc * loc))>
 ==>
 <(rel * SAME) --> (loc * ocr)> &/ <(sample * C1) --> (loc * ocr)>
  <=>
 <(sample * B1) --> (loc * ocr)>
```

Then, by substituting

```
<(rel * SAME) --> (loc * ocr)> &/
 <(sample * C1) --> (loc * ocr)>
```

for

```
<(sample * B1) --> (loc * ocr)>. :|:
```

the operation `<({SELF} * clap) --> ^action>` would be executed, by using the previously trained contingency involving $B1$ and `^clap`.

Similarly, the following situation

```
<(rel * OPPOSITE) --> (loc * ocr)>. :|:
<(sample * C1) --> (loc * ocr)>. :|:
G! :|:
```

would lead to the operation `<({SELF} * wave) --> ^action>` would be executed, using similar derivations. Especially the following network will be important in the derivation:

```
<(OPPOSITE * (C1 * B2)) --> (ocr * (ocr * ocr))> &&
 <(rel * (sample * sample)) --> (loc * (loc * loc))>
 ==>
 <(rel * OPPOSITE) --> (loc * ocr)> &/ <(sample * C1) --> (loc * ocr)
    >
  <=>
 <(sample * B2) --> (loc * ocr)>
```
